# Predictors of 1-year mortality in patients on prolonged mechanical ventilation after surgery in intensive care unit: a multicenter, retrospective cohort study

**DOI:** 10.1186/s12871-020-0942-0

**Published:** 2020-02-21

**Authors:** Yueming Sun, Shuangling Li, Shupeng Wang, Chen Li, Gang Li, Jiaxuan Xu, Hongzhi Wang, Fei Liu, Gaiqi Yao, Zhigang Chang, Yalin Liu, Meixia Shang, Dongxin Wang

**Affiliations:** 1 Department of Critical Care Medicine, Peking University First Hospital, Beijing, 100034 China; 2 Department of Critical Care Medicine, China-Japan Friendship Hospital, Beijing, 100029 China; 3 Department of Critical Care Medicine, Beijing Cancer Hospital, Beijing, 100142 China; 4 Department of Critical Care Medicine, Peking University Third Hospital, Beijing, 100191 China; 5 Department of Critical Care Medicine, National Center of Gerontology, Beijing Hospital, Beijing, 100730 China; 6 Department of Biostatistics, Peking University First Hospital, Beijing, 100034 China

**Keywords:** Intensive care unit, Prolonged mechanical ventilation, Postoperative patients, 1-year mortality

## Abstract

**Objectives:**

The requirement of prolonged mechanical ventilation (PMV) is associated with increased medical care demand and expenses, high early and long-term mortality, and worse life quality. However, no study has assessed the prognostic factors associated with 1-year mortality among PMV patients, not less than 21 days after surgery. This study analyzed the predictors of 1-year mortality in patients requiring PMV in intensive care units (ICUs) after surgery.

**Methods:**

In this multicenter, respective cohort study, 124 patients who required PMV after surgery in the ICUs of five tertiary hospitals in Beijing between January 2007 and June 2016 were enrolled. The primary outcome was the duration of survival within 1 year. Predictors of 1-year mortality were identified with a multivariable Cox proportional hazard model. The predictive effect of the ProVent score was also validated.

**Results:**

Of the 124 patients enrolled, the cumulative 1-year mortality was 74.2% (92/124). From the multivariable Cox proportional hazard analysis, cancer diagnosis (hazard ratio [HR] 2.14, 95% confidence interval [CI] 1.37–3.35; *P* < 0.01), no tracheostomy (HR 2.01, 95% CI 1.22–3.30; *P* < 0.01), enteral nutrition intolerance (HR 1.88, 95% CI 1.19–2.97; *P* = 0.01), blood platelet count ≤150 × 10^9^/L (HR 1.77, 95% CI 1.14–2.75; *P* = 0.01), requirement of vasopressors (HR 1.78, 95% CI 1.13–2.80; *P* = 0.02), and renal replacement therapy (HR 1.71, 95% CI 1.01–2.91; *P* = 0.047) on the 21st day of mechanical ventilation (MV) were associated with shortened 1-year survival.

**Conclusions:**

For patients who required PMV after surgery, cancer diagnosis, no tracheostomy, enteral nutrition intolerance, blood platelet count ≤150 × 10^9^/L, vasopressor requirement, and renal replacement therapy on the 21st day of MV were associated with shortened 1-year survival. The prognosis in PMV patients in ICUs can facilitate the decision-making process of physicians and patients’ family members on treatment schedule.

## Introduction

Mechanical ventilation (MV) is a frequently applied invasive technique to patients admitted in the intensive care unit (ICU). Although MV is usually seen as a life-saving strategy, it has a strong potential to worsen the prognosis with prolonged use. According to the 2005 consensus of the National Association for Medical Direction of Respiratory Care (NAMDRC), prolonged mechanical ventilation (PMV) is defined as the requirement of MV for more than 6 h daily and that lasts for more than 21 consecutive days [1]. The requirement of PMV is associated with increased medical care demand and expenses [2, 3], high early and long-term mortality [4, 5], and worse life quality [6–9]. Therefore, it is necessary to evaluate the prognosis in PMV patients in ICUs to facilitate the decision making process of the physicians and patients’ family members on treatment schedule in the ICUs.

In 2008, Carson and colleagues [10] developed a mortality-prediction model (Prognosis for Prolonged Ventilation, i.e., ProVent score) to estimate the risk of 1-year mortality in patients receiving MV for at least 21 days. They used four factors including age, platelet count, use of vasopressors, and requirement of renal replacement therapy to calculate the ProVent score. Another study showed only low platelet count on the 21st day of MV as a predictor of 1-year mortality in patients requiring PMV in a medical ICU in Korea [11]. However, in a mixed ICU study in France, use of vasopressors, and requirement of renal replacement therapy are predictors of 1-year mortality in patients requiring PMV [12]. Therefore, due to the inconsistent findings, further studies are needed to evaluate this further. In addition, no study has yet to demonstrate the predictors of 1-year mortality in patients requiring PMV in ICU after surgery. The purpose of this study was to analyze the predictors of 1-year mortality in patients requiring PMV in ICU after surgery.

## Material and methods

This was a multicenter, retrospective cohort study.

### Ethics approval and consent to participate

The study protocol was approved by the Ethics Committee of Peking University First Hospital (2017[1422]). As the study was purely observational and retrospective in nature, the Ethics Committee agreed to exempt the written informed consent. However, all enrolled patients or their family members had to verbally agree to participate in the long-term follow-up before the data collection.

### Patients

Patients who required MV after surgery in the ICUs in five tertiary hospitals (Peking University First Hospital, Peking University Third Hospital, China-Japan Friendship Hospital, Beijing Cancer Hospital, and Beijing Hospital) in Beijing between January 2007 and June 2016 were screened. The inclusion criteria were those aged 18 years or older, admitted to the ICU after surgery, and received MV for at least 21 consecutive days. Patients who met any of the following criteria were excluded: acute or chronic neuromuscular diseases (such as Guillain-Barré syndrome, muscular dystrophy, amyotrophic lateral sclerosis, or myasthenia gravis), requirement of invasive MV before ICU admission, or incomplete clinical information.

### Patient and public involvement

The research question and outcome were not informed by patients’ priorities, experience, and preference. Patients didn’t involve in the design of this study. Patients didn’t involve in the recruitment to and conduct of the study. The results wasn’t disseminated to study participants. All enrolled patients or their family members verbally agree to participate in the long-term follow-up before the data collection and researchers thanked them.

### Clinical data collection

Data were collected using the medical record system in each hospital. Baseline data included demographic characteristics (age and gender), preoperative comorbidities, the New York Heart Association (NYHA) functional classification, liver and renal function tests, radio- or chemotherapy within 6 months, reason for surgery, location of surgery. Others included the scores of Sequential Organ Failure Assessment (SOFA) [13] and Acute Physiology and Chronic Health Evaluation (APACHE) II within the first 24 h after ICU admission.

On the 21st day of MV, data including platelet count, requirement of renal replacement therapy (currently or within 48 h), use of vasopressors (currently or within 48 h), mode of nutritional supplementation, state of consciousness, presence of multi-drug resistant bacterial infection, and performance of tracheostomy were collected. Other data including the duration of MV, length of stay in ICU and hospital, and ICU and hospital mortalities, were recorded. The 3-month, 6-month, and 1-year survival status after day 21 of MV were documented from the medical record system or at follow-up. The record was checked every three months.

### Statistical analysis

Patients were divided into survivor and non-survivor groups according to the 1-year survival status. Continuous variables were presented as means ± standard deviation (SD) or median (interquartile range [IQR]) and compared with unpaired t test or Mann-Whitney U test. Categorical variables were presented as numbers (%) and compared using χ^2^ or Fisher’s exact test. The 95% confidence interval (CI) of mortality in patients was estimated by the Bootstrap method (the number of execution samples was 1000).

Factors in association with 1-year survival were analyzed using the Cox proportional hazard model. Factors with number of events > 10 were screened. Ten factors were identified at univariate analysis. Those with *P* < 0.10 were included in the multivariate model after testing for collinearity. Collinearity of factors were tested using linear regression. Independent factors in association with 1-year survival were identified using a backward stepwise method. The effects of the combination of our multivariate factors and the ProVent score in predicting 1-year survival in patients with PMV were compared using the receiver operator characteristic (ROC) curves and the area under the curve (AUC). All tests were two-sided. *P* values ≤0.05 were considered statistically significant. Statistical analysis was performed with the SPSS 21.0 software package (Inc, Chicago, IL).

## Results

### Patient recruitment and baseline characteristics

**Fig. 1:**
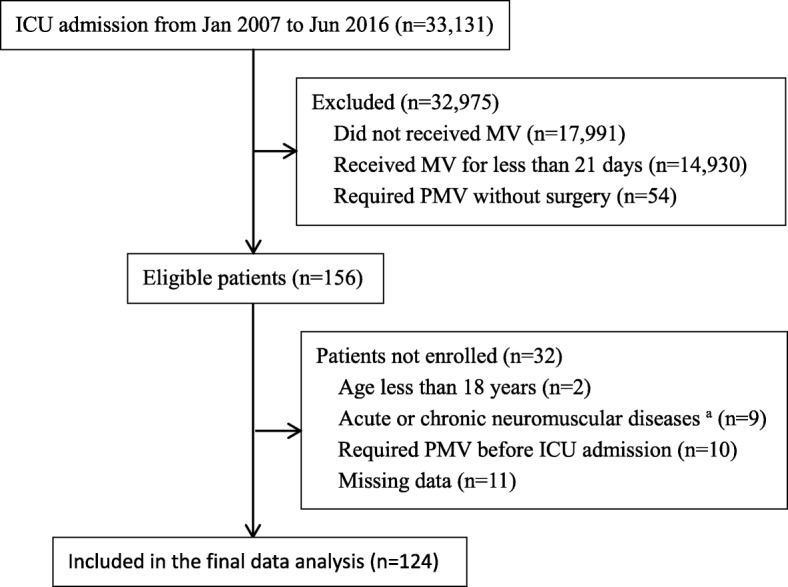
Flow chart of the study. ICU, intensive care unit. MV, mechanical ventilation. PMV, prolonged mechanical ventilation. ^a^Included Guillain-Barré syndrome, muscular dystrophy, amyotrophic lateral sclerosis and myasthenia gravis

From January 2007 to June 2016, 33,131 patients who were admitted to ICUs in the 5 tertiary hospitals were screened for study participation. Of these, 156 patients (0.5%) required PMV after surgery (incidence of PMV was 0.5%); and 124 (0.4%) fulfilled the inclusion/exclusion criteria and were included in the final statistical analysis (Fig. 1).

Of 124 patients included in the study, mean (±SD) age was 66.5 (±16.5) years, 72.6% (90/124) were male, and 35.5% (44/124) underwent cancer surgery (Table 1).

**Table 1:** Baseline characteristics

	Total (*N* = 124)	Non-survivors (*N* = 92)	Survivors (*N* = 32)	*P* value
Age (y)	66.5 ± 16.5	67.8 ± 15.8	62.8 ± 18.1	0.138
Male sex	90 (72.6)	67 (72.8)	23 (71.9)	0.917
Preoperative comorbidities				
Central nervous system	24 (19.4)	19 (20.7)	5 (15.6)	0.535
Stroke	16 (12.9)	13 (14.1)	3 (9.4)	0.760
Cephalomeningitis	5 (4.0)	3 (3.3)	2 (6.3)	0.603
Parkinsonism	2 (1.6)	2 (2.2)	0 (0.0)	> 0.999
Intracranial aneurysm	1 (0.8)	1 (1.1)	0 (0.0)	> 0.999
Dementia	1 (0.8)	1 (1.1)	0 (0.0)	> 0.999
Respiratory system	37 (29.8)	27 (29.4)	10 (31.3)	0.839
Smoking ^a^	31 (25.0)	22 (23.9)	9 (28.1)	0.636
Tuberculosis	5 (4.0)	5 (5.4)	0 (0.0)	0.326
Asthma	4 (3.2)	3 (3.3)	1 (3.1)	> 0.999
COPD	4 (3.2)	2 (2.2)	2 (6.3)	0.274
Pulmonary heart disease	1 (0.8)	1 (1.1)	0 (0.0)	> 0.999
Circulatory system	79 (63.7)	59 (64.1)	20 (62.5)	0.869
Hypertension	66 (53.2)	49 (53.3)	17 (53.1)	0.989
Coronary heart disease	27 (21.8)	22 (23.9)	5 (15.6)	0.328
Arrhythmia	14 (11.3)	8 (8.7)	6 (18.8)	0.122
Cardiomyopathy	1 (0.8)	1 (1.1)	0 (0.0)	> 0.999
Metabolic and immune system	35 (28.2)	30 (32.6)	5 (15.7)	0.066
Diabetes	24 (19.4)	21 (22.8)	3 (9.4)	0.097
Hyperlipemia	5 (4.0)	5 (5.4)	0 (0.0)	0.326
Thyroid disease	2 (1.6)	2 (2.2)	0 (0.0)	> 0.999
Rheumatoid arthritis	2 (1.6)	1 (1.1)	1 (3.1)	0.451
Gout	1 (0.8)	1 (1.1)	0 (0.0)	> 0.999
Systemic lupus erythematosus	1 (0.8)	0 (0.0)	1 (3.1)	0.258
NYHA functional classification				0.058
I	63 (50.8)	42 (45.7)	21 (65.6)	
II	34 (27.4)	30 (32.6)	4 (12.5)	
III	17 (13.7)	11 (12.0)	6 (18.8)	
IV	10 (8.1)	9 (9.8)	1 (3.1)	
Abnormal liver function ^b^	5 (4.0)	4 (4.4)	1 (3.1)	> 0.999
Abnormal renal function ^c^	5 (4.0)	4 (4.4)	1 (3.1)	> 0.999
Radio−/chemotherapy in 6 months	4 (3.2)	2 (2.2)	2 (6.3)	0.274
Cancer surgery	44 (35.5)	40 (43.5)	4 (12.5)	0.002
Location of surgery				0.015
Intra-thoracic	45 (36.3)	39 (42.4)	6 (18.8)	
Intra-abdominal	40 (32.3)	30 (32.6)	10 (31.3)	
Others ^d^	39 (31.5)	23 (25.0)	16 (50.0)	
Preoperative ASA classification				0.270
I	10 (8.1)	9 (9.8)	1 (3.1)	
II	63 (50.8)	43 (46.7)	20 (62.5)	
III	22 (17.7)	16 (17.4)	6 (18.8)	
IV	29 (23.4)	24 (26.1)	5 (15.6)	
Urgent surgery	37 (29.8)	27 (29.3)	10 (31.3)	0.839
ICU admission for new onset POCs	31 (25.0)	18 (19.6)	13 (40.6)	0.018
Scores on ICU admission				
SOFA score	7.4 ± 3.3	7.6 ± 3.4	6.5 ± 2.5	0.051
APACHE II score	20.0 ± 7.3	20.9 ± 7.7	19 ± 5.7	0.210

Results are presented as mean ± SD and numbers (%)

COPD, chronic obstructive pulmonary diseases. NYHA, the New York Heart Association. ICU, intensive care unit. POCs, postoperative complications. SOFA, sequential organ failure assessment. APACHE, acute physiology and chronic health evaluation

^a^Smoking for more than 10 cigarettes per day for more than 1 year, including current or past smokers

^b^Serum alanine transaminase or aspartate transaminase higher than 5 times of the normal upper limit

^c^Defined as glomerular filtration rate (GFR) < 60 ml/min/1.73 m^2^

^d^Include neurosurgery, thyroid surgery and orthopedic surgery

### Status on day 21 of mechanical ventilation

Compared with 1-year non-survivors, those who survived underwent more tracheostomy (*P* < 0.01) and enteral nutrition (*P* = 0.01), suffered less from low platelet-count (≤150 × 10^9^/L; *P* = 0.02), and required less renal replacement therapy (*P* = 0.03) (Table 2).

**Table 2:** Patients’ situation on the 21st day of mechanical ventilation

Situation on 21st day of MV	Total (*N* = 124)	Non-survivors (*N* = 92)	Survivors (*N* = 32)	*P* value
No tracheostomy	31 (25.0)	30 (32.6)	1 (3.1)	< 0.01
Consciousness (GCS = 15)	74 (59.7)	54 (43.6)	20 (62.5)	0.71
Drug-resistant bacteria infection	63 (50.8)	51 (55.4)	12 (37.5)	0.08
MDR	50 (40.3)	39 (42.4)	11 (34.4)	0.43
XDR	13 (10.5)	12 (13.0)	1 (3.1)	0.18
Intolerance of enteral nutrition^a^	43 (34.7)	38 (41.3)	5 (15.6)	0.01
Platelet ≤150 × 10^9^/L	49 (39.5)	42 (45.7)	7 (21.9)	0.02
On vasopressors	41 (33.1)	33 (35.9)	8 (25.0)	0.26
On renal replacement therapy	24 (19.4)	22 (23.9)	2 (6.3)	0.03

Results are presented as mean ± SD, median (interquartile range) and numbers (%)

MV, mechanical ventilation. GCS, Glasgow coma scale. MDR, multi-drug-resistant. XDR, Extensively drug-resistant

^a^Included total parenteral and combined enteral-parenteral nutrition

### Outcomes

Of all included patients, when counted from the 21st day of MV, the median duration of MV was 35 days (interquartile range [IQR] 27–61). The median length of stay in ICU and hospital were 44.0 (IQR 31.3–73.5) and 65.5 (IQR, 41.3–117.3) days, respectively. The mortality rates were 67.7% [84/124] (95% confidence interval [CI] 59.7–75.8%) in ICU, 71.0% [88/124] (95% CI 62.9–79.0%) in hospital, and 74.2% [92/124] (95% CI 66.9–81.5%) at 1 year. The main cause of death was multiple organ failure syndrome (MODS) induced by septic shock (67.0% [59/124]).

### Factors associated with 1-year survival

Factors with number of events > 10 were screened. Ten factors were identified at univariate analysis (*P* < 0.10) (Table 3).

**Table 3:** Risk factors in association with 1-year survival (univariate Cox Proportional Hazard analyses)

Risk factors	N	HR (95% CI) ^a^	*P* value
Baseline characteristics			
Age (y)			
< 50	20	1.000	
50–64	25	1.06 (0.51–2.19)	0.89
≥ 65	79	1.48 (0.81–2.69)	0.20
Male sex	90	1.05 (0.66–1.66)	0.85
Stroke	16	1.09 (0.60–1.95)	0.78
Smoking ^b^	31	1.18 (0.73–1.90)	0.51
Hypertension	66	1.09 (0.72–1.64)	0.69
Coronary heart disease	27	0.87 (0.54–1.40)	0.56
NYHA functional classification			
I + II	97	1.000	
III + IV	27	1.01 (0.62–1.66)	0.97
Diabetes	24	1.13 (0.89–1.44)	0.33
Cancer diagnosis	44	1.20 (0.98–1.48)	0.08
Location of surgery			
Others ^c^	39	1.000	
Intra-thoracic/abdominal	85	1.73 (1.08–2.78)	0.02
SOFA score on ICU admission	124	1.09 (1.01–1.16)	0.02
APACHE II score on ICU admission	124	1.03 (1.00–1.06)	0.037
Situation on the 21st day of MV			
No tracheostomy	31	2.67 (1.70–4.17)	< 0.001
Consciousness (GCS = 15)	74	0.79 (0.52–1.20)	0.27
MDR/XDR bacterial infection	63	1.53 (1.02–2.32)	0.042
Intolerance of enteral nutrition ^d^	43	1.90 (1.25–2.90)	< 0.01
Platelet ≤150 × 10^9^/L	49	1.97 (1.30–2.97)	< 0.01
On vasopressors	41	1.70 (1.11–2.61)	0.02
On renal replacement therapy	24	1.59 (0.97–2.61)	0.068

HR, hazard ratio. CI, confidence interval. NYHA, the New York Heart Association. ICU, intensive care unit. SOFA, sequential organ failure assessment. APACHE, acute physiology and chronic health enquiry. MV, mechanical ventilation. GCS, Glasgow coma scale. MDR, multi-drug-resistant. XDR, Extensively drug-resistant

^a^Factors with number of events > 10 were included. ^b^ Smoking of more than 10 cigarettes per day for more than 1 year, including current or previous smokers. ^c^ Included neurosurgery, thyroid surgery, and orthopedic surgery. ^d^ Included total parenteral and combined enteral-parenteral nutrition

Of these, SOFA score was excluded because of collinearity with APACHE II score. The remaining nine factors were included in the multivariate Cox proportional hazard model. Six independent factors were identified to be associated with 1-year survival. Cancer surgery (hazard ratio [HR] 2.14, CI 1.37–3.35; *P* < 0.01), no tracheostomy (HR 2.01, 95% CI 1.22–3.30; *P* < 0.01), enteral nutrition intolerance (HR 1.88, 95% CI 1.19–2.97; *P* = 0.01), platelet count ≤150 × 10^9^/L (HR 1.77, 95% CI 1.14–2.75; P = 0.01), vasopressor requirement (HR 1.74, 95% CI 1.11–2.74; *P* = 0.02), and renal replacement therapy (HR 1.71, 95% CI 1.01–2.91; *P* = 0.047) on the 21st day of MV were associated with shortened 1-year survival (Table 4).

**Table 4:** Predictors of 1-year survival (multivariate Cox Proportional Hazard analyses)

Predictors	Univariate analysis	Multivariate Analysis ^a^	
	*P* value	HR (95% CI)	*P* value
Cancer diagnosis	0.083	2.14 (1.37–3.35)	< 0.01
Intra-thoracic/abdominal surgery (vs. others) ^b^	0.023	–	–
SOFA score on ICU admission ^c^	0.019	–	–
APACHE II score on ICU admission	0.037	–	–
No tracheostomy on day 21 of MV	< 0.001	2.01 (1.22–3.30)	< 0.01
MDR/XDR bacterial infection on day 21 of MV	0.042	–	–
Intolerance of enteral nutrition on day 21 of MV ^d^	0.003	1.88 (1.19–2.97)	0.01
Platelet ≤150 × 10^9^/L on day 21 of MV	0.001	1.77 (1.14–2.75)	0.01
On vasopressors on day 21 of MV	0.015	1.74 (1.11–2.74)	0.02
On renal replacement therapy on day 21 of MV	0.068	1.71 (1.01–2.91)	0.047

HR, hazard ratio. CI, confidence interval. ICU, intensive care unit. SOFA, sequential organ failure assessment. APACHE, acute physiology and chronic health evaluation. MV, mechanical ventilation. MDR, multi-drug-resistant. XDR, Extensively drug-resistant

^a^Factors with P < 0.10 and number of outcomes > 10 were included in the multivariate model (Backward). ^b^Included neurosurgery, thyroid surgery, and orthopedic surgery. ^c^ Not included because of collinearity with APACHE II score. ^d^ Included total parenteral and combined enteral-parenteral nutrition

### Comparison between the combination of our multivariate factors and the ProVent score in predicting 1-year mortality

The combination of multivariate factors could be used in predicting 1-year survival in these patients with PMV after surgery (area under curve [AUC] 0.81 [95% CI 0.72–0.89]). The ROC curve was based on the combination of all the 6 independent predictors of death.

**Fig. 2:**
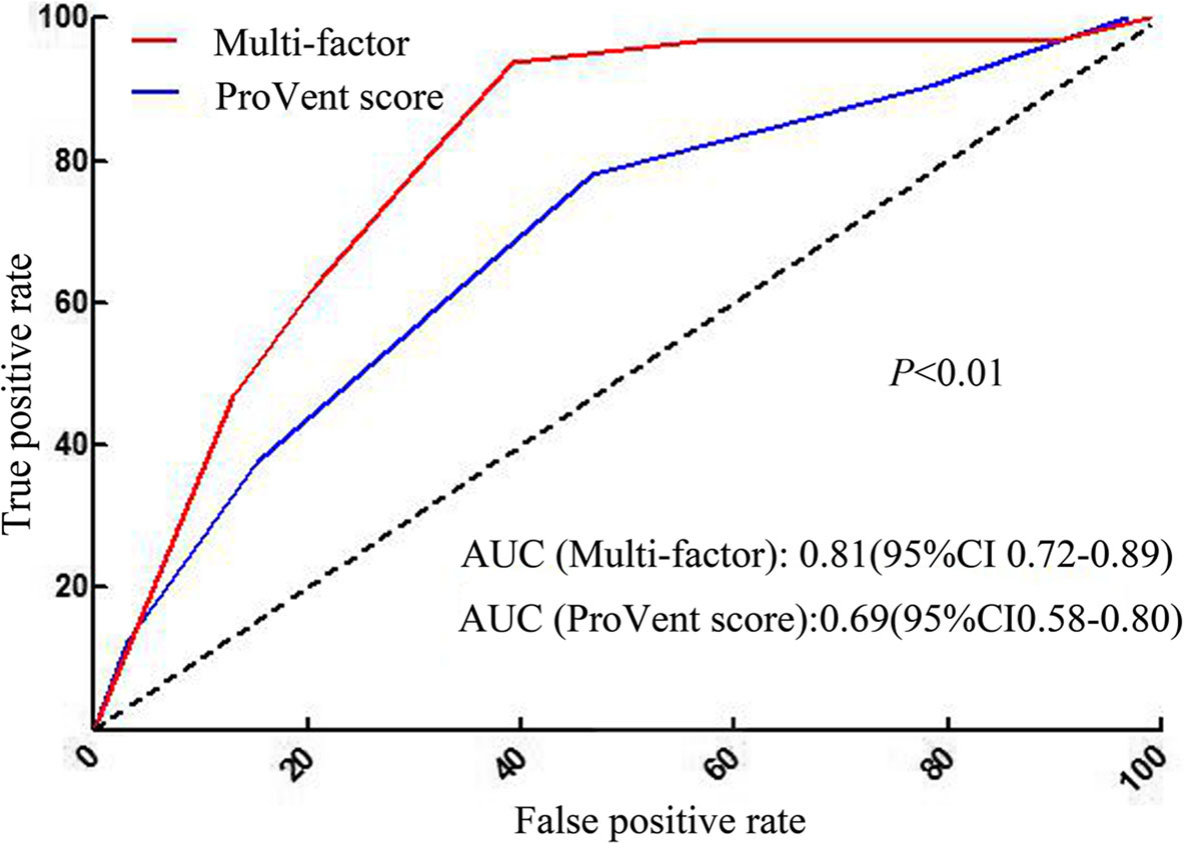
Comparison between the combination of multivariate factors and the ProVent Score in predicting 1-year mortality

There was significant difference between the combination of our multivariate factors and the ProVent score in predicting 1-year mortality in these patients with PMV after surgery (area under curve [AUC] 0.81 [95% CI 0.72–0.89] vs. 0.69 [95% CI 0.58–0.80]; *P* < 0.01) (Fig. 2).

## Discussion

Despite a small incidence of postoperative PMV of only 0.5%, among patients, the mortality were as high as 67.7 and 71.0% in the ICU and hospital, respectively. In studies on patients with PMV in ICUs, the reported 1-year mortality varied from 48 to 67% [11, 12, 14], which is lower than that in our cohort (74.2%). Patients on PMV after surgery had worse 1-year prognosis than patients in mixed and medical ICUs. From the China National Committee on Aging data (http://www.cncaprc.gov.cn/contents/37/69715.html), people older than 60 years will be about 248 million by 2020 (17% of the present population in China). Along with an aging population is the progression in medical technology and therapeutic theory, the number of PMV patients will also be increasing in China with time. Thus, it is of great clinical significance to evaluate the prognosis of PMV patients in ICUs.

Our results showed that, for patients requiring PMV after surgery, cancer surgery, together with no tracheostomy, enteral nutrition intolerance, platelet count ≤150 × 10^9^/L, use of vasopressors and renal replacement therapy on the 21st day of MV was associated with shortened 1-year survival. Since it is a retrospective study, it is impossible to know whether the factors associated with mortality are markers of severity or determinants of death.

A study showed that low platelet count, use of vasopressors, and requirement of renal replacement therapy on day 21 of MV are predictors of 1-year mortality in PMV patients in a mixed ICU in the United States [14], which is similar to our finding. The same study showed that age was a predictor of 1-year mortality in PMV patients, but this was not demonstrated in our study; this might have been caused by the different study populations. In critically ill patients, thrombocytopenia is usually caused by severe infections, side effect of medications, and myelosuppression among others; which is regarded as a sign of illness aggravation [15]. Requirement of vasopressors implies an unstable circulation, which is associated with higher occurrence of multiple organ dysfunction syndrome (MODS) [16]. In patients with sepsis and MODS, requirement of vasopressors is also associated with increased 1-year and 5-year mortality [17]. Requirement of renal replacement therapy on the 21st day of MV is usually caused by renal failure, and is also regarded as a sign of poor prognosis [12].

A study showed that the overall 1-year survival rate in PMV cancer patients was 14.3% [13], which was poorer generally, than that in patients with other comorbidities [18]. After cancer surgery, PMV patients showed poor prognosis, which could be attributed to the cancer itself as well as the development of cancer recurrence/metastasis after surgery [19, 20].

In a study involving 429 patients which evaluated hospital and long-term outcome after tracheostomy for respiratory failure, the results showed that those who were weaned off MV and placed on tracheostomy tubes had lower 3-year mortality than ventilator-dependent patients (*P* < 0.001) [21]. In the present study, the patients not inserting tracheostomy might often meant with high risk of death, and have increased the use of sedatives and opioids, maybe dependence on MV ultimately, which was associated with prolonged 1-year mortality.

Critically ill patients on MV are at risk of underfeeding and progressive malnutrition, and this population often receives less than the required energy and protein [22]. Enteral nutrition (EN) is preferred over parenteral nutrition (PN) because it is more physiological and less likely to result in hepatobiliary dysfunction and electrolyte imbalance [23]. In addition, when compared with EN, use of PN has been linked to higher incidence of infection, impaired wound healing, and gastrointestinal bleeding [24].

The multivariate Cox proportional model can be used as a prognostic assessment tool for critically ill patients after surgery in the future. Clinicians should not only pay attention to platelet count, use of vasopressors, and the need for renal replacement on the 21st day of MV in patients, but also to assess whether patients have malignant tumors, need tracheostomy, and enteral nutritional support.

The area under curve for the combination of our multivariate factors was more than the area for the ProVent score. There was significant difference between the ProVent score and the combination of our multivariate factors in predicting 1-year survival using ROC curves, however, the sample size was relatively small and the comparison might be unconvincing.

This study had major limitations. First, the sample size was relatively small. Patients requiring PMV after surgery had a small sample size, with a percentage of only 0.5% in our study. A larger sample size is needed to develop a more accurate predictive model. Secondly, our study retrospectively analyzed patients’ data over a long period. Clinical practice and, thus, patient characteristics might have changed during that period, which made it lack of validation.

## Conclusions

For patients requiring PMV after surgery, cancer diagnosis, no tracheostomy, enteral nutrition intolerance, low platelet count, and dependence on vasopressors and renal replacement therapy on the 21st day of MV were associated with worse 1-year survival. The prognosis in PMV patients in ICUs can facilitate the decision that making process of the physicians and patients’ family members on treatment schedule.

## Data Availability

The datasets generated and/or analysed during the current study are not publicly available due to when individual privacy could be compromised but are available from the corresponding author on reasonable request.

## Abbreviations

APACHE: acute physiology and chronic health evaluation
CI: confidence interval
COPD: chronic obstructive pulmonary diseases
GCS: Glasgow coma scale
HR: hazard ratio
ICU: intensive care unit
MDR: multi-drug-resistant
MV: mechanical ventilation
NYHA: the New York Heart Association
POCs: postoperative complications
SOFA: sequential organ failure assessment
XDR: Extensively drug-resistant
